# Inequities in Unmet Oral Care Needs after a Swedish Subsidization Reform: An Intersectional Analysis

**DOI:** 10.1177/23800844241305109

**Published:** 2024-12-19

**Authors:** C. Anticona, A.L. Suominen, J.L. Bastos, P. Lif Holgerson, P.E. Gustafsson

**Affiliations:** 1 Department of Epidemiology and Global Health, Umeå University, Umeå, Sweden; 2 Department of Odontology, Umeå University, Umeå, Sweden; 3 Institute of Dentistry, University of Eastern Finland, Kuopio, Finland; 4 Oral and Maxillofacial Teaching Unit, Kuopio University Hospital, Kuopio, Finland; 5 Faculty of Health Sciences, Simon Fraser University, Burnaby, Canada; 6 Department of Odontology, Section of Pediatric Dentistry, Umeå University, Umeå, Sweden

**Keywords:** dental public health, dental care, health care inequities, universal health insurance, health care reform, intersectional framework

## Abstract

**Introduction:**

The main strategy to achieve equal provision of oral care in Sweden has been to offer partial subsidies for the adult population. However, their effects on unmet oral care needs (UOCNs) have not been extensively assessed.

**Objective:**

This study used an intersectionality framework to examine 1) the overall frequency of UOCNs, 2) single-indicator inequities, and 3) intersectional inequities in total UOCNs and financial-related UOCNs (FUOCNs) in Sweden before and after implementation of a partial subsidization reform in 2008.

**Methods:**

Data from 12 national surveys conducted over 2004 to 2018 were divided into 3 periods: prereform (2004 to 2007), early postreform (2008 to 2011), and late postreform (2012 to 2018). The analytic sample consisted of 98,177 respondents aged 24 to 84 y. Changes in the prevalence of UOCNs were estimated by inferential statistics. Single-indicator and intersectional inequities were examined by intersectional analysis of individual heterogeneity and discriminatory accuracy, across 48 strata defined by gender, age, educational level, income, and immigrant status.

**Results:**

The prevalence of total UOCNs and FUOCNs decreased significantly early after the reform, followed by a slight rebound. Relative inequities increased by education, income, and immigrant status after the reform and decreased for age. The discriminatory accuracy for both types of UOCNs was moderate and improved marginally but significantly with the inclusion of the intersectional strata. Most intersectional strata showed greater FUOCN inequities after the reform.

**Conclusions:**

Contrary to expected, larger inequities in FUOCNs were identified in most intersectional strata after the reform. The moderate discriminatory accuracy suggested that Sweden could benefit from future strategies to foster equity that are universal but proportionately more intense among the intersectional strata with greater inequities (proportionate universalism).

**Knowledge Transference Statement:**

This analysis highlighted the benefit of adopting the principle of proportionate universalism as a strategy to reduce unmet oral care needs in Sweden. This would mean implementing universal strategies and specific support measures for the most vulnerable social groups as a future oral care policy change in Sweden.

## Introduction

The principle of equity in the provision of oral care implies that all individuals should have an equal opportunity to use oral care services, according to their needs and regardless of other factors (Raine et al. 2016). This notion is supported by the recognition that oral health is a fundamental human right and should be a public responsibility. Health systems have thus been urged to provide comprehensive oral care to all residents (World Health Organization 2020). However, only a few countries (e.g., Thailand and Brazil) have adopted universal oral health care models where a package of essential services is mostly publicly funded for the entire population. Most countries provide partial coverage, which is generally lower than that for general health care (Winkelmann et al. 2023).

Research assessing the effects of subsidization policies (Somkotra and Detsomboonrat 2009; Raittio et al. 2015; Hernández-Vásquez et al. 2019; Kim et al. 2019) has consistently shown increased utilization of oral care services but absent or unclear benefits for those with the greatest need or for the most marginalized groups. Indeed, the most vulnerable groups tend to use oral care less, irrespective of the need or costs implicated (Winkelmann et al. 2022). Even in contexts with extensive public funding of oral care, individual-level indicators of social vulnerability (e.g., income and race) persist as strong determinants of oral care utilization (Raittio et al. 2015; Herkrath et al. 2018).

Sweden provides an interesting example of policy approaches to advancing oral health and care equity, with the Swedish Dental Care Act explicitly aiming for equitable oral health and oral care (Statens Offentliga Utredningar 2021). The strategy to achieve this goal has traditionally relied on partial subsidization for adults, which has changed over time. Initially, treatment cost coverage was 75% in the 1970s but reduced to 30% by 1999, alongside deregulation of oral care prices (Astrom et al. 2021). Governmental evaluations of these changes led to a new reform in 2008. This reform sought to enhance equity by promoting preventive care and affordable services for those with major oral care needs. The reform introduced annual allowances intended to cover primarily standard oral examinations or other preventive services partially and strengthened a high-cost protection scheme (not discussed in this study). Allowances consisted of 150 SEK (13 EUR in 2024) to all adults, with double the amount for the younger patients (24 to 29 y) and older ones (≥75 y; Statens Offentliga Utredningar 2021). Further details are in Appendix File 1.

The partial subsidies system is still in place but with scant scientific evidence on its equity effects. Most recently, oral care inequities (i.e., preventable and unjust health-related disparities between groups) have drawn the attention of multiple sectors of the Swedish society, including authorities, media, and the general public. This is in response to the increasing social gap for oral diseases, services utilization, and unmet oral care needs (UOCNs) (Astrom et al. 2021). UOCNs are a relevant inequity indicator, expressing the divergence between the care required and received (Torppa-Saarinen et al. 2021).

Considering the lack of evidence on the impact of the reform and public concern over rising health inequities in Sweden, further research examining the 2008 reform is essential to ground future policy. Relatedly, a novel research approach in social inequities, based on an intersectionality perspective, has recently gained traction in oral health (Muirhead et al. 2020). Intersectionality-based research examines how interrelated social processes, embodied by individuals, shape living conditions and health outcomes in dynamic and contextually specific ways (Muirhead et al. 2020). Accordingly, it provides a more comprehensive and nuanced mapping of inequities than traditional approaches assessing a specific indicator at a time (Merlo et al. 2023). Our previous study exploring UOCNs in Swedish adults over 2004 to 2021 showed that while the most vulnerable social groups, such as low-income and low-educated young immigrants, reported the highest risk for UOCNs, other privileged groups, such as high-income young natives with low education, reported a high risk (Anticona et al. 2023). However, it did not assess UOCN inequities over time or the impact of the 2008 reform. The aim of this study was to apply an intersectionality framework to assess total UOCN (TUOCN) and financial-related UOCN (FUOCN) inequities among Swedish adults before and after the 2008 reform. The specific aims were to examine to which degree the reform affected 1) population-average UOCN prevalence, 2) single-indicator UOCN inequities, and 3) intersectional UOCN inequities.

## Methods

### Design and Ethics

This study used a repeated cross-sectional design. Data came from 12 Health on Equal Terms surveys conducted in Sweden annually between 2004 and 2018, excluding 2015, 2016 (the outcome of interest was not included), and 2017 (no survey was conducted). The 2018 survey was included considering that the annual subsidy was implemented during the survey administration months and was therefore unlikely to affect the UOCN perception. Three study periods were considered—prereform (P1), 2004 to 2007; early postreform (P2), 2008 to 2011; and late postreform (P3), 2012 to 2018—based on studies assessing the effects of a Finnish reform, which concluded that the initial reduction of oral care inequities did not remain in a late postreform period (Raittio et al. 2015; Raittio et al. 2016).

The Health on Equal Terms survey is performed by the Public Health Agency of Sweden, and it collects self-reported information on general and oral health, health care utilization, lifestyle, and living conditions of individuals aged 16 to 84 y. The response rates have decreased over time, from 60.8% in 2004 to 42.1% in 2018. The total sample consisted of 117,189 respondents (response rate, 47.0%). From this sample, individuals aged 16 to 23 y (*n* = 9,966, 8.5%) were excluded because they were not affected by the reform. Additionally, individuals with missing information on the outcomes or social indicators were excluded (*n* = 9,046, 8.4%). The analytic sample was 98,177 (91.6% of all respondents aged ≥24 y). This study conforms to STROBE reporting guidelines and was approved by the Swedish Ethical Review Authority (approval 2021-02398).

### Variables

Two outcomes were considered: TUOCNs and FUOCNs, with the latter being a subset of the former. While TUOCN assesses the perceived care need that is untreated, FUOCN specifically captures financial barriers, representing a subjective nonchosen unmet need (Allin et al. 2010). FUOCNs were expected to be directly affected by the reform, as it introduced partial subsidies to reduce oral care costs.

TUOCN was assessed by the answer to the following question: “Have you during the last 3 months believed yourself to be in need of dental care but refrained from seeking care?” If the answer was yes, the respondent had a TUOCN (coding = 1; otherwise, coding = 0).

FUOCN was measured by the follow-up question: “What was/were the reason(s) why you did not seek dental care?” with 5 multiple-choice response options: “the problem cleared up,” “financial reasons,” “declined to go (fear of dentists),” “did not have time,” and “other reason.” Respondents selecting the “financial reasons” option were categorized as having FUOCNs (coding = 1; otherwise, coding = 0).

The explanatory variables were 5 social indicators retrieved from Statistics Sweden, categorized and coded as follows: gender, defined by the proxy variable of sex (woman = 1, man = 0); age (24 to 44 y = 2, 45 to 64 y = 1, 65 to 84 y = 0); education (low, <3 y of high school = 1; high, ≥3 y of high school = 0); immigrant status, determined as having immigrated to Sweden at any point in life (immigrant = 1, native = 0); and individual disposable income (low, less than median = 1; high, median or higher = 0) adjusted for inflation based on official consumer price indices. All categories were subsequently cross-classified to create a multicategorical variable comprising 48 mutually exclusive intersectional strata. The reference stratum consisted of native men aged ≥65 y with high education and high income. No covariates were included to avoid overadjustment for potential intervening variables.

### Analysis

Descriptive analyses for the entire sample and each period were conducted. This was followed by a series of inferential analyses of the impact of the reform on UOCN inequities. Generalized linear models with binomial family and log link function were used for TUOCNs and FUOCNs (equations in Appendix File 2). Results are reported as prevalence ratios (PRs) with 95% CIs.

Changes in the prevalence of reporting TUOCNs and FUOCNs (aim 1) across periods were estimated with the prereform period as reference (P2 vs. P1 and P3 vs. P1). A crude model without adjustments and an adjusted model including the social indicators mentioned previously were estimated.

Assessment of changes in single-indicator inequities (aim 2) and intersectional inequities (aim 3) in UOCNs was conducted by an intersectionality-informed analysis of individual heterogeneity and discriminatory accuracy (Merlo et al. 2023). For the individual heterogeneity component, TUOCNs and FUOCNs were assessed in 2 consecutive models, separately for each period. Model 1 considered single-indicator inequities, and model 2 replaced the indicators with the intersectional strata variable comprising 48 cross-classified strata (Appendix Table 3). Changes in inequities across periods were estimated for each indicator (model 1) and intersectional stratum (model 2) by the relative PR changes between periods by group × period interaction terms (PR interaction: P2 vs. P1, P3 vs. P2, and P3 vs. P1). For illustrative purposes, intersectional strata were also classified by the pattern of change in PR across periods (Appendix File 3): little or no change, decreased, rebounding, persistent increased, and delayed increased inequities. Seven strata with too small sample size could not be classified (TUOCNs, *k* = 3; FUOCNs, *k* = 4).

The discriminatory accuracy (DA) component estimated the accuracy with which the indicators in the model discriminated individuals with and without UOCNs, as an estimate of the magnitude of inequities considering within-group variation (Merlo et al. 2023). DA of the separate indicators and the intersectional strata was estimated by calculating the area under the receiver operating characteristic curve (AUC) for each period by models 1 and 2, as previously described. The results were interpreted per a classification proposed elsewhere (Axelsson Fisk et al. 2021). The change of AUC value (ΔAUC) was calculated to 1) quantify the improvement in DA between models 1 and 2 by considering intersectional strata instead of single indicators and 2) to quantify changes in single and intersectional inequities between periods.

Sensitivity analyses included 1) assessment of the annual AUC values for model 2 and 2) quantitative bias analysis of the selection bias related to the explanatory variables and FUOCNs (Fox et al. 2021). All analyses were conducted with Stata 14.0 (StataCorp).

## Results

### Population Characteristics

The distribution of social indicators evolved in the study population over time, with increasing proportions of high income, high education, older age, and immigrant status but with comparable gender distribution. The prevalence of TUOCNs and FUOCNs decreased over time. However, the social patterning of UOCNs remained similar, with a numerically higher prevalence of both types of UOCNs among people with low education, low income, younger age, and immigrant status but with a comparable prevalence between gender categories (Table 1).

**Table 1.** Prevalence of Total and Financial-Related UOCNs across Social Indicators and Study Periods.

	Prereform (2004 to 2007)			Early Postreform (2008 to 2011)			Late Postreform (2012 to 2018)		
	Total	Total UOCNs	Financial-Related UOCNs	Total	Total UOCNs	Financial-Related UOCNs	Total	Total UOCNs	Financial-Related UOCNs
Total sample	23,626 (100.0)	4,386 (18.6)	3,142 (13.3)	33,978 (100.0)	5,096 (15.0)	3,343 (9.8)	40,573 (100.0)	5,530 (13.6)	3,364 (8.3)
**Social indicator**									
Gender									
Man	10,723 (45.4)	1,984 (18.5)	1,360 (12.7)	15,253 (44.9)	2,263 (14.8)	1,433 (9.4)	18,681 (46.0)	2,601 (13.9)	1,522 (8.2)
Woman	12,903 (54.6)	2,402 (18.6)	1,782 (13.8)	18,725 (55.1)	2,833 (15.1)	1,910 (10.2)	21,892 (54.0)	2,929 (13.4)	1,842 (8.4)
Education									
High	12,705 (53.8)	2,220 (17.5)	1,543 (12.1)	19,508 (57.4)	2,765 (14.2)	1,783 (9.1)	24,855 (61.3)	3,081 (12.4)	1,769 (7.1)
Low	10,921 (46.2)	2,166 (19.8)	1,599 (14.6)	14,470 (42.6)	2,331 (16.1)	1,560 (10.8)	15,718 (38.7)	2,449 (15.6)	1,595 (10.2)
Age, y									
65 to 84	3,882 (16.4)	418 (10.8)	269 (6.9)	8,413 (24.8)	768 (9.1)	440 (5.2)	13,718 (33.8)	1,285 (9.4)	720 (5.3)
45 to 64	10,309 (43.6)	1,649 (16.0)	1,134 (11)	14,257 (42.0)	2,017 (14.2)	1,309 (9.2)	15,601 (38.5)	2,175 (13.9)	1,318 (8.5)
24 to 44	9,435 (39.9)	2,319 (24.6)	1,739 (18.4)	11,308 (33.3)	2,311 (20.4)	1,594 (14.1)	11,254 (27.7)	2,070 (18.4)	1,326 (11.8)
Income									
High	8,540 (36.1)	1,091 (12.8)	665 (7.8)	15,900 (46.8)	1,726 (10.9)	934 (5.9)	22,431 (55.3)	2,301 (10.3)	1,150 (5.1)
Low	15,086 (63.9)	3,295 (21.8)	2,477 (16.4)	18,078 (53.2)	3,370 (18.6)	2,409 (13.3)	18,142 (44.7)	3,229 (17.8)	2,214 (12.2)
Immigrant									
No	22,387 (94.8)	3,975 (17.8)	2,846 (12.7)	29,098 (85.6)	3,817 (13.1)	2,430 (8.4)	35,570 (87.7)	4,339 (12.2)	2,588 (7.3)
Yes	1,239 (5.2)	411 (33.2)	296 (23.9)	4,880 (14.4)	1,279 (26.2)	913 (18.7)	5,003 (12.3)	1,191 (23.8)	776 (15.5)

Data are presented as *n* (%).

Abbreviation: UOCNs, unmet oral care needs.

### Prevalence of UOCNs Pre- and Postreform

The prevalence of UOCNs was compared across periods (Appendix Table 2). In the adjusted analyses, the prevalence of TUOCNs and FUOCNs decreased significantly early postreform (P1 to P2, PR [95% CI]: 0.86 [0.83, 0.89] for TUOCNs; 0.82 [0.78, 0.85] for FUOCNs). This decrease did not continue into the late postreform period, showing a slight rebound instead (P1 to P3, PR [95% CI]: 0.90 [0.87, 0.93] for TUOCNs; 0.83 [0.79, 0.87] for FUOCNs].

### Single-Indicator UOCN Inequities Pre- and Postreform

Relative TUOC and FUOC inequities by single indicators are reported by PR (95% CI). The magnitude of inequities was different for each indicator (Appendix Table 2). The largest TUOCN inequities were seen for age, specifically for the youngest group (PR, 2.20; 2.11, 2.30) and for immigration status (PR, 1.85; 1.79, 1.92). Smaller inequities were found for income (PR, 1.76; 1.71, 1.82), followed by education (PR, 1.19; 1.16, 1.23), while gender inequities were unsubstantial (PR, 0.99; 0.96, 1.02). FUOCN inequities followed a similar pattern, although they were larger for all indicators. The largest inequities in FUOCNs were seen for age (youngest group; PR, 2.65; 2.50, 2.80) and income (PR, 2.35; 2.26, 2.46).

Changes in single-indicator inequities are reported in Table 2, including the main effects (inequities within period) and the interaction terms by period (changes in inequities between periods). The UOCN-related inequities displayed a reconfiguration, especially by education and age, following the reform. The initial education gap (PR, 1.13) remained unchanged in P2 but increased significantly in P3 (PR, 1.25; PR interaction [95% CI], 1.10 [1.02, 1.20]). Conversely, the gap for the group aged 24 to 44 y displayed a constant decrease postreform (PR, 2.28 in P1 and 1.96 in P3; PR interaction, 0.86 [0.77, 0.97]). Meanwhile the inequities for the group aged 45 to 64 y showed a negligible increase in P2 (PR, 1.49 in P1 and 1.55 in P2; PR interaction, 1.04 [0.91, 1.19]) that rebounded in P3 (PR, 1.49).

**Table 2.** UOCNs by Social Indicator in the Study Periods: Main and Interaction Effects.

	Prevalence Ratio (95% CI)					
	Main Effects			Interaction Effects		
	Prereform	Early Postreform	Late Postreform	Early Post- vs. Prereform	Late vs. Early Postreform	Late Post- vs. Prereform
**Total UOCNs**						
Gender						
Man	1	1	1	1	1	1
Woman	1.00 (0.95, 1.10)	1.02 (0.97, 1.07)	0.96 (0.91, 1.00)	1.01 (0.94, 1.09)	0.94 (0.87, 1.01)	0.95 (0.88, 1.02)
Education						
High	1	1	1	1	1	1
Low	1.13 (1.07, 1.19)	1.13 (1.08, 1.19)	1.25 (1.19, 1.32)	1.00 (0.93, 1.10)	1.10 (1.03, 1.18)	1.10 (1.02, 1.20)
Age, y						
65 to 84	1	1	1	1	1	1
45 to 64	1.49 (1.34, 1.64)	1.55 (1.43, 1.68)	1.49 (1.39, 1.59)	1.04 (0.91, 1.19)	0.96 (0.86, 1.06)	1.00 (0.89, 1.12)
24 to 44	2.28 (2.10, 2.51)	2.24 (2.07, 2.42)	1.96 (1.84, 2.09)	0.98 (0.86, 1.10)	0.88 (0.80, 0.97)	0.86 (0.77, 0.97)
Income						
High	1	1	1	1	1	1
Low	1.70 (1.61, 1.82)	1.71 (1.63, 1.81)	1.74 (1.65, 1.82)	1.00 (0.92, 1.09)	1.01 (0.94, 1.09)	1.01 (0.93, 1.10)
Immigrant						
No	1	1	1	1	1	1
Yes	1.87 (1.72, 2.03)	1.99 (1.89, 2.11)	1.95 (1.84, 2.07)	1.06 (0.97, 1.18)	0.97 (0.90, 1.05)	1.04 (0.94, 1.16)
**Financial-related UOCNs**						
Gender						
Man	1	1	1	1	1	1
Woman	1.10 (1.01, 1.16)	1.08 (1.01, 1.15)	1.03 (0.97, 1.10)	0.99 (0.91, 1.09)	0.95 (0.87, 1.04)	0.94 (0.86, 1.04)
Education						
High	1	1	1	1	1	1
Low	1.20 (1.13, 1.29)	1.18 (1.11, 1.26)	1.43 (1.33, 1.52)	0.97 (0.89, 1.07)	1.20 (1.11, 1.26)	1.18 (1.07, 1.29)
Age, y						
65 to 84	1	1	1	1	1	1
45 to 64	1.59 (1.39, 1.80)	1.75 (1.58, 1.95)	1.61 (1.47, 1.75)	1.11 (0.93, 1.30)	0.92 (0.79, 1.05)	1.01 (0.87, 1.18)
24 to 44	2.65 (2.35, 3.00)	2.69 (2.43, 2.98)	2.24 (2.06, 2.44)	1.01 (0.86, 1.18)	0.83 (0.72, 0.95)	0.84 (0.72, 0.98)
Income						
High	1	1	1	1	1	1
Low	2.11 (1.94, 2.09)	2.27 (2.11, 2.44)	2.38 (2.22, 2.54)	1.08 (0.96, 1.19)	1.04 (0.94, 1.15)	1.13 (1.01, 1.25)
Immigrant						
No	1	1	1	1	1	1
Yes	1.88 (1.69, 2.09)	2.24 (2.09, 2.40)	2.13 (1.98, 2.29)	1.19 (1.05, 1.35)	0.95 (0.86, 1.05)	1.13 (0.99, 1.29)

Interaction effects for each social indicator were calculated to indicate the prevalence ratio changes among the 3 study periods.

Abbreviation: UOCNs, unmet oral care needs.

Income and immigration gaps displayed negligible increases over time. Gender inequities were insubstantial and unchanged across periods.

Comparable but considerably more pronounced patterns were found for FUOCN inequities. Gaps increased significantly for education (P1 to P3: PR, 1.20 to 1.43; PR interaction, 1.18 [95% CI, 1.07, 1.29]), income (PR, 2.11 to 2.38 ; PR interaction, 1.13 [1.01, 1.25]), and immigration (PR, 1.88 to 2.13; PR interaction, 1.19 [1.05, 1.35]). The opposite was observed for the age gap, which decreased significantly for the youngest group (P1 to P3: PR, 2.65 to 2.24; PR interaction, 0.84 [0.72, 0.98]).

The bias analysis showed that the bias-adjusted estimates were consistently larger for all estimates under the assumption of a 10–percentage point selection bias by the outcome FUOCNs. Additionally, the weak gender estimates changed direction, with the disadvantaged group changing from men to women. The degree of selection bias was larger postreform than prereform (Appendix File 5).

### Intersectional UOCN Inequities Pre- and Postreform

**Figure 1.**
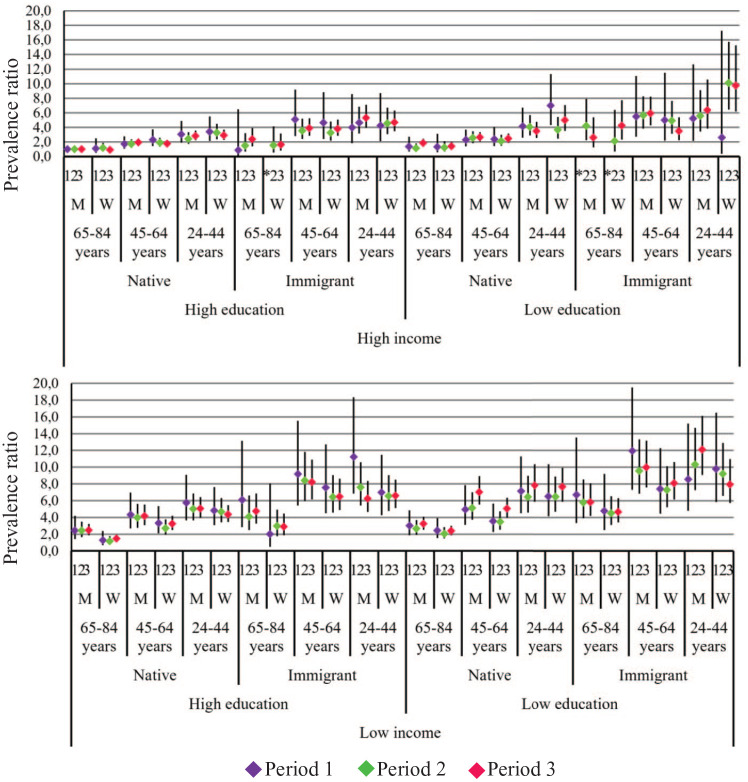
Relative inequities of total unmet oral care needs for 48 intersectional strata in 3 study periods. Estimates are prevalence ratios and 95% CIs in each study period consecutively, from periods 1 to 3. The reference stratum consists of native men aged ≥65 y with high education and high income. *Missing estimate. M, man; W, woman.

**Figure 2.**
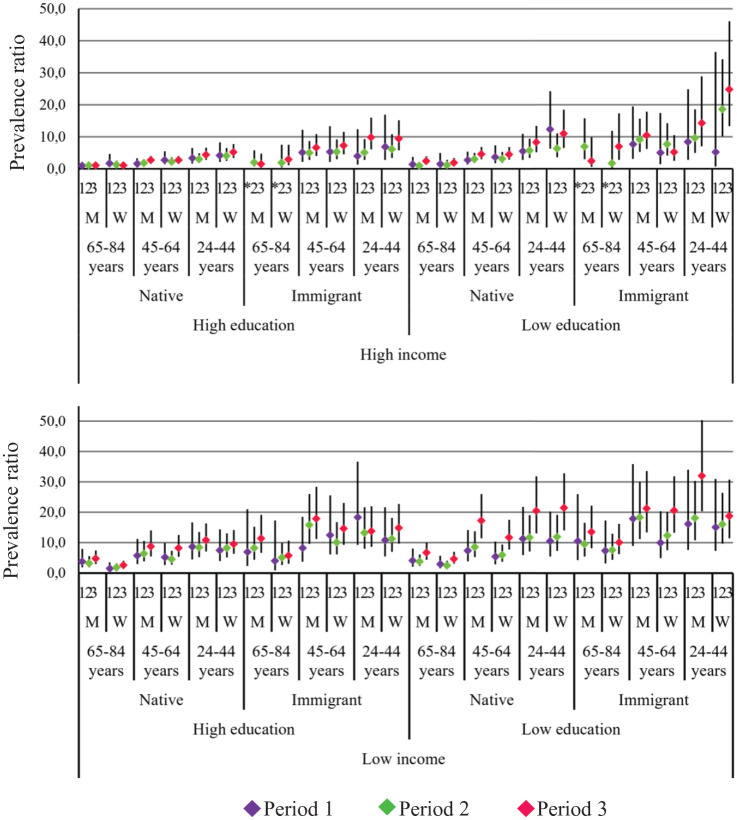
Relative inequities of financial-related unmet oral care needs for 48 intersectional strata in 3 study periods. Estimates are prevalence ratios and 95% CIs in each study period consecutively, from periods 1 to 3. The reference stratum consists of native men aged ≥65 y with high education and high income. *Missing estimate. M, man; W, woman.

Intersectional inequities in TUOCNs and FUOCNs are reported by the stratum-specific PR (95% CI) in Appendix Table 3 and illustrated in Figures 1 and 2. The largest TUOCN inequities (PR >8.00 in all periods) were observed among 2 strata comprising immigrant men aged <65 y with low education and income. Conversely, the smallest inequities (PR >1.20 in all periods) were displayed by native women aged >64 y with high education and income. Similar patterns were found for FUOCNs, although with larger inequities among the most socially disadvantaged strata.

Changes in inequities across the 3 periods are displayed in Appendix Table 2 and Appendix Figure 1. For TUOCNs, we observed a similar number of intersectional strata experiencing decreased inequities (*k* = 23) and increased inequities (persistent, *k* = 8; delayed, *k* = 10). The patterns of change were notably distinct for FUOCNs, with the majority of strata experiencing persisting (*k* = 23) or delayed (*k* = 15) increased inequities and only 4 experiencing decreased inequities. Of the 4 strata displaying decreased inequities for TUOCNs and FUOCNs, 3 consisted of high-income Sweden-born women—specifically, Sweden-born elderly women. One stratum was young immigrant men with high education and low income. Conversely, the strata with persisting increased inequities for both types of UOCNs (*k* = 6) were all formed by men aged <65 y and almost all (*k* = 5) with low education. Due to the small sample size, period comparisons were significant for only 4 strata (Appendix Table 3).

### Discriminatory Accuracy

For TUOCNs, the DA estimated for models 1 (separate indicators) and 2 (intersectional strata) was moderate in all periods (AUC, 0.64 to 0.67), although with a marginally improved DA for model 2 as compared with model 1 (ΔAUC, 0.01 for all periods). The DA improved slightly but significantly from P1 to P2 in both models, with no change from P2 to P3 (Table 3).

**Table 3.** Discriminatory Accuracy of Single Indicators and Intersectional Model in the Study Periods.

	Model Estimates, AUC (95% CI)			Model Comparisons, *P* Value		
	Prereform	Early Postreform	Late Postreform	Early Post- vs. Prereform	Late vs. Early Postreform	Late Post- vs. Prereform
**Total UOCNs**						
Model 1: single indicators	0.64 (0.63, 0.65)	0.66 (0.65, 0.67)	0.66 (0.65, 0.66)	<0.01	0.35	<0.05
Model 2: intersectional strata	0.65 (0.64, 0.66)	0.67 (0.66, 0.67)	0.67 (0.66, 0.67)	<0.01	0.63	<0.01
ΔAUC ^a^	0.01	0.01	0.01			
**Financial-related UOCNs**						
Model 1: single indicators	0.66 (0.65, 0.67)	0.69 (0.68, 0.70)	0.70 (0.69, 0.71)	<0.01	0.55	<0.01
Model 2: intersectional strata	0.67 (0.66, 0.68)	0.70 (1.69, 0.71)	0.71 (0.69, 0.71)	<0.01	0.79	<0.01
ΔAUC ^a^	0.01	0.01	0.01			

Abbreviations: AUC, area under the receiver operating characteristic curve; UOCNs, unmet oral care needs.

a Model comparison refers to model 2.

The DA for FUOCNs was consistently higher when compared with TUOCNs, for both models in all periods and with the highest estimate found for model 2 in P3 (AUC, 0.71). The DA improved significantly from P1 to P2 in both models, with no change from P2 to P3. The annual changes in AUC values calculated in the sensitivity analysis showed an increasing trend before the implementation of the reform for TUOCNs and FUOCNs (Appendix Fig. 2).

## Discussion

The novel findings from this analysis demonstrated that the population-average prevalence of TUOCNs and FUOCNs decreased immediately postreform but did not continue to decrease in the subsequent years and rather showed signs of a slight rebound. Moreover, analyses suggested that single-indicator and intersectional inequities in TUOCNs, and particularly FUOCNs, increased early postreform and remained high in the following years. Taken together, these findings suggest that the reform brought about 1) a moderate but potentially waning reduction in population-level UOCNs and 2) a complex FUOCN-related increase in inequities.

The decreasing prevalence of TUOCNs and FUOCNs observed between 2004 and 2018 aligned with European statistics (Winkelmann et al. 2022) suggesting that the observed positive trend in Sweden might be, at least partly, attributed to broader secular trends. Despite this, oral care has consistently been the most frequent type of care for which European people report FUOCNs, with a generally higher prevalence in countries with low public oral care coverage (European Commission-Eurostat 2022b).

Although a decreasing prevalence of both types of UOCNs was observed in the descriptive analysis, adjusted analysis indicated a slight rebound between the early and late postreform periods. This suggests that any positive effect of the reform to reduce UOCNs was short-lived. Universal financial measures in other countries have also led to an improvement in oral care utilization (e.g., increased dental visits) but smaller effects on reducing inequities (Somkotra and Detsomboonrat 2009; Raittio et al. 2015; Kim et al. 2019; Kim and Kawachi 2021).

The gap in TUOCNs and FUOCNs increased by education, income, and migration postreform but decreased for the youngest group. An assessment of self-rated oral health inequities after the 2008 reform showed that the education and income gaps increased postreform, with less clear changes in age and migration gaps (Anticona et al. 2024). This suggests the growing relevance of education and income inequities for oral care and health indicators postreform. Similarly, income and education were related to persistently high frequencies of UOCNs in Finland despite a major subsidization reform in 2001 to 2002 (Torppa-Saarinen et al. 2021), and migration remains an important predictor for UOCNs in Europe, with nonnationals having a 15% higher probability of reporting UOCNs as compared with nationals (European Commission-Eurostat 2022a).

The intersectional analysis showed first of all, that the DA for TUOCNs and FUOCNs increased gradually postreform and improved slightly but significantly after including the intersectional strata in the model. This illustrates that the intersectional inequities were able to discriminate between individuals with and without UOCNs, with marginally greater differences than the single indicators. However, sensitivity analysis of the annual changes in DA showed an increase before the reform, suggesting that the increasing inequities also were influenced by secular trends and not merely attributed to the reform. Consequently, future strategies to reduce UOCNs might benefit from being universal but proportionately more intense among the intersectional strata with larger UOCN inequities (Merlo et al. 2023).

Second, inequities changed differently for TUOCNs and FUOCNs postreform. Almost half the intersectional strata showed decreased TUOCN inequities, but only 4 showed decreased FUOCN inequities. Of these 4 strata, 3 consisted of socially advantaged Sweden-born women of high income. Contrarily, all strata that consisted of participants with low education and low income showed increased FUOCN inequities. This illustrates the detrimental and favorable cumulative effects of multiple social disadvantages and advantages (Crenshaw 1989). Additionally, the interaction of social advantages and disadvantages in each stratum was critical to shape the risk of increased inequities postreform; for example, neither being native nor having high income seemed to protect low-educated middle-aged men from experiencing persistent increased FUOCN inequities. Similarly, high income did not protect young immigrant men if they had low education. Noteworthy is that the period comparison within each intersectional stratum relied on descriptive patterns of change, as statistical significance could not be estimated due to the small size of the intersectional strata. These findings align with the single-indicators analysis, suggesting that the relevance of education for oral health–related inequities increased postreform. Moreover, the likelihood of reporting UOCNs among Swedish adults is highly heterogenous between and within the groups defined by gender, age, education, income, and migration status. Finally, the findings underscore the value of an intersectional approach in identifying which groups benefited the most and the least from the reform and to further examine the underlying drivers.

There are several possible explanations for the limited effect of financial measures on equitable oral care. Out-of-pocket costs and limited cost coverage affect social groups differently. While well-off groups can experience financial hardship, impoverished groups might forgo oral care, particularly preventive services (Winkelmann et al. 2022). This issue is exacerbated by the price liberalization of oral care and a strong presence of the private sector (Raittio et al. 2015), as seen in Sweden. This is exemplified by the annual subsidy, which covered only 25% of an oral examination cost for adults aged 25 to 64 y in 2008. By 2014, it barely covered 20% due to the rising prices, particularly in private clinics (Statens Offentliga Utredningar 2021). Furthermore, utilization of private care is disproportionally higher among well-off groups who can afford and demand higher-quality care. This could mean that the public services that are mostly used by the poor eventually show declines in service quality (Palència et al. 2014). An equal distribution of oral care providers and facilities is another challenge. In Sweden, a growing staffing shortage in the public sector has resulted in restrictions to access regular preventive care, particularly in the Northern regions (Tandvårds- och läkemedelsförmånsverket 2023). Broader factors contributing to persistent and wider health inequities include the growing importance of nonmaterial resources (e.g., social support) and personal characteristics (e.g., cognitive ability), which are highly socially differentiated in welfare countries (Mackenbach 2012).

Taken together, the analysis suggested that the 2008 subsidization reform, while being followed by a transitory decrease in UOCNs, did not lead to reduced FUOCN inequities. The intersectional analysis showed greater UOCN inequities postreform, with the greatest vulnerability to UOCNs among the groups with multiple social disadvantages. Thus, more effective policies based on the principle of proportionate universalism might be beneficial. This would mean adopting universal care systems, including specific support mechanisms for the most marginalized groups. This is also supported by the moderately increasing DA of the models (Merlo et al. 2023). Although the findings might mirror the situation of European countries with similar oral care systems, we express caution about generalizing to other countries due to the context-specific nature of the outcomes.

The main strengths of this study were the large sample and the novel intersectional approach. Limitations include using a repeated cross-sectional design without a control condition, making it difficult to attribute any observed changes to the reform. The social indicators were categorized as required for the intersectional analysis but with the risk of losing variation in the outcomes. UOCN was self-assessed with subsequent risk for information bias. Moreover, the potential effect of selection bias was assessed, suggesting that the reported estimations of the magnitude of and increase in inequities from the pre- to postreform periods were likely underestimated. Finally, UOCN was used as an indicator of care utilization, as more specific data (e.g., number of dental visits) were not available in the Health on Equal Terms survey. Further research based on care utilization registers would contribute to a more comprehensive evaluation.

## Conclusions

The prevalence of TUOCNs and FUOCNs decreased early postreform but showed signs of rebound later. Education-, income-, and immigration-related inequities became more relevant postreform, except for the youngest group. Unexpectedly, larger FUOCN inequities were found in most intersectional strata. Future research should explore the drivers of the persistent or increased UOCN inequities postreform and identify effective equity-promoting oral health policies.

## Author Contributions

C. Anticona, contributed to conception, data acquisition, analysis, and interpretation, drafted and critically revised the manuscript; A.L. Suominen, J.L. Bastos, P. Lif Holgerson, contributed to conception, data interpretation, drafted and critically revised the manuscript; P.E. Gustafsson, contributed to conception, design, data acquisition, analysis, and interpretation, drafted and critically revised the manuscript; All authors gave final approval and agree to be accountable for all aspects of the work.

## Supplemental Material

sj-docx-3-jct-10.1177_23800844241305109 – Supplemental material for Inequities in Unmet Oral Care Needs after a Swedish Subsidization Reform: An Intersectional Analysis Supplemental material, sj-docx-3-jct-10.1177_23800844241305109 for Inequities in Unmet Oral Care Needs after a Swedish Subsidization Reform: An Intersectional Analysis by C. Anticona, A.L. Suominen, J.L. Bastos, P. Lif Holgerson and P.E. Gustafsson in JDR Clinical & Translational Research

sj-pptx-1-jct-10.1177_23800844241305109 – Supplemental material for Inequities in Unmet Oral Care Needs after a Swedish Subsidization Reform: An Intersectional Analysis Supplemental material, sj-pptx-1-jct-10.1177_23800844241305109 for Inequities in Unmet Oral Care Needs after a Swedish Subsidization Reform: An Intersectional Analysis by C. Anticona, A.L. Suominen, J.L. Bastos, P. Lif Holgerson and P.E. Gustafsson in JDR Clinical & Translational Research

sj-pptx-2-jct-10.1177_23800844241305109 – Supplemental material for Inequities in Unmet Oral Care Needs after a Swedish Subsidization Reform: An Intersectional Analysis Supplemental material, sj-pptx-2-jct-10.1177_23800844241305109 for Inequities in Unmet Oral Care Needs after a Swedish Subsidization Reform: An Intersectional Analysis by C. Anticona, A.L. Suominen, J.L. Bastos, P. Lif Holgerson and P.E. Gustafsson in JDR Clinical & Translational Research
