# Supplementary material for: Inequities in Unmet Oral Care Needs after a Swedish Subsidization Reform: An Intersectional Analysis
Source: JDR Clin Trans Res. 2024 Dec 19;10(4):416–26. doi: 10.1177/23800844241305109 (PMC12402522; doi:10.1177/23800844241305109)
Supplement: sj-docx-3-jct-10.1177_23800844241305109 – Supplemental material for Inequities in Unmet Oral Care Needs after a Swedish Subsidization Reform: An Intersectional Analysis [file sj-docx-3-jct-10.1177_23800844241305109.docx]

**Supplemental appendix**

**Title:** Inequities in unmet oral care needs after a Swedish subsidization reform: An intersectional analysis

**Authors**

Cynthia Anticona

Department of Epidemiology and Global Health, Umeå University, SE-90187 Umeå, Sweden.

Department of Odontology, Umeå University, SE-90185 Umeå,

Email: [cynthia.anticona@umu.se](mailto:cynthia.anticona@umu.se)

Anna Liisa Suominen

Institute of Dentistry, University of Eastern Finland, Kuopio, Finland.

Oral and Maxillofacial Teaching Unit, Kuopio University Hospital, Kuopio, Finland

Email: :liisa.suominen@uef.fi

João Luiz Bastos

Faculty of Health Sciences, Simon Fraser University, Burnaby, BC Canada.

Email: [jbastos@sfu.ca](mailto:jbastos@sfu.ca)

Pernilla Lif Holgerson

Department of Odontology, Section of Pediatric Dentistry, Umeå University, SE-90185 Umeå, Sweden. Email: [pernilla.lif@umu.se](mailto:pernilla.lif@umu.se)

Per E Gustafsson

Department of Epidemiology and Global Health, Umeå University, 901 87, Umeå, Sweden

Email: [per.e.gustafsson@umu.se](mailto:per.e.gustafsson@umu.se)

**Appendix File 1**

**Partial subsidies and high-cost protection scheme for oral care**

The oral health reform implemented in Sweden in 2008 comprised two main components : i) the introduction of annual subsidies to partially cover the cost of oral care services and ii) the strengthening of an existing high-cost protection scheme.

The universal subsidies for all adults consisted of a fixed annual sum that varied according to the patient´s age. Individuals aged 30–64 years received 150 SEK while younger (aged 24–29 years) and older adults (aged ≥75 years) received 300 SEK. In 2017, the age limit for the higher subsidy was lowered from 75 to 65 years. In April 2018, the subsidy was doubled for all groups. As of 2024, the subsidy amounts to 300 SEK (26 EUR) per year for individuals aged 30–64 years and 600 SEK (52 EUR) per year for individuals aged 24–29 or ≥ 65 years ([Statens Offentliga Utredningar 2021](#_ENREF_4)).

The high-cost protection scheme was initially introduced in 1999 for “non-basic care” procedures, primarily prosthetics and orthodontics, with a deductible amount of 3500 SEK. In 2002, the high-cost protection was extended for individuals aged ≥ 65 years , allowing those individuals with costs for prosthetic measures exceeding 7700 SEK to receive a compensation for the entire excess amount (excluding certain material costs and specific implant treatments). In 2008, the high-cost protection subsidy was further strengthened, providing a subsidy of 50% for all individuals whose treatments costs exceeded 3000 SEK and 85% for costs above 15000 SEK. No further modifications to the high-cost protection have been made after the 2008 reform ([Statens Offentliga Utredningar 2021](#_ENREF_4)).

National reports evaluating the effects of the 2008 reform indicate that the specific objectives have only been partially met. The utilization of preventive care increased minimally (2%) in the overall population (from 57% per year in 2009 to 59% per year in 2011) and predominantly among the youngest group (20–24 years). Furthermore, the high-cost protection failed to benefit those with lowest incomes and largest oral care needs ([Riksrevisionen 2012](#_ENREF_3)). The primary reason appears to be the deductible amount (3000 SEK), which is too high for those with significant oral care needs and low incomes. This issue is likely exacerbated by the constant increase of price of oral health services, particularly in the private sector ([Riksrevisionen 2012](#_ENREF_3)).

**Appendix File 2**

**Mathematical equation for a generalized linear model with binomial family and log link**

A series of generali zed linear models with binomial family and log link function was conducted to assess the impact of the reform on inequities in total and financial-related unmet oral care needs (TUOCN and FUOCN), addressing the three study aims.

**Aim 1**

Changes in the overall prevalence of reporting TUOCN or FUOCN across the three periods were examined using the pre-reform period (P1) as reference. The crude model was:

$$Y=a+b_{1}P_{2}+ b_{2}P_{3}+ \varepsilon$$

The same model adjusted for social indicators was:

$$Y=a+b_{1}P_{2}+b_{2}P_{3} + b_{3}I_{1}+b_{4}I_{2}+b_{5}I_{3}+b_{6}I_{4}+b_{7}I_{5} + \varepsilon$$

Where P represents *period* (period 1= P1; period 2=P2; period 3=P3) and I represents *social indicator* (I1= gender, I2= age, I3= education, I4= income, I5= migration status).

**Aim 2**

Changes in single-indicators inequities in TUOCN and FUOCN were examined by periods, including both the main effects and the interaction terms by period. The model to examine the main effects by period and social indicators was:

$$Y=a + b_{1}I + \varepsilon$$

The model to examine the interaction terms by period and social indicators was:

$$Y=a+b_{1}I+b_{2}P+b_{3}I*P+ \varepsilon$$

**Aim 3**

Changes in intersectional inequities in TUOCN and FUOCN were examined by periods, including both the main effects and the interaction terms by period. The model for the main effects was:

$$Y=a + b1S + \varepsilon$$

The model to examine the interaction terms by period was:

$$Y=a + b_{1}S + b_{2}P+b_{3}S*P+ \varepsilon$$

Where S represent the *intersectional stratum* and P represents *period* (period 1= P1; period 2=P2; period 3=P3).

**Appendix File 3**

**Classification of the intersectional strata based on the pattern of change in inequities in unmet oral care needs (Prevalence ratio) across the study periods: period 1 (P1), period 2 (P2) and period 3 (P3)**

1. Little or no change (decrease or increase < 2% for both comparisons: P2 vs. P1 and P3 vs. P2)

2. Decreased inequities (decrease > 2% for P3 vs. P1)

3. Rebounding inequities (increase > 2% for P2 vs. P1and decrease > 2% for P3 vs. P2)

4. Persistent increased inequities (increase > 2% for both comparisons: P2 vs. P1 and P3 vs. P1)

5. Delayed increased inequities (any decrease for P2 vs. P1 and increase > 2% for P3 vs. P2).

**Appendix File 4**

**Sensitivity analysis**

The discriminatory accuracy (AUC) values for the model including the intersectional strata were calculated to assess the annual changes. As illustrated in Appendix Figure 2, the AUC values have an increasing trend before 2008. This suggest increasing inequities even before the implementation of the reform (2008). However, the large confidence intervals, that in most cases overlap with adjacent point estimates suggest little statistical support for changes between the individual years.

**Appendix File 5**

**Quantitative bias analysis to assess the potential effect of selection bias**

The risk of selection bias was considered due to the decreasing response rate of the survey over the study period (2004–2018) and the underrepresentation of some intersectional groups. A simple quantitative bias analysis ([Fox et al. 2021](#_ENREF_2)) was therefore conducted to assess the effects of the bias on the observed results.

**Methods**

**Selection proportions for the explanatory variables.**

Selection proportions of the participation rate in the three study periods (pre-reform = 2004–2007; early post-reform = 2008–2011 and late post-reform = 2012–2018) by each of the five social indicators (gender, age, education, income and migration) were estimated from participation rate data in the annual “Health on Equal Terms” survey reported by Statistics Sweden. This information was however only available for some years; thus, three specific years were selected to represent the participation rate for each of the periods:

- Participation rates in 2004 ([Statistiska Centralbyrån 2004](#_ENREF_5) ) = period 1

- Participation rates in 2010 ([Statistiska Centralbyrån 2010](#_ENREF_6)) = period 2

- Participation rates in 2018 ([Statistiska Centralbyrån 2018](#_ENREF_7)) = period 3

Available data on the participation rates per social indicator was dichotomized as a requirement to conduct the bias analysis: gender (woman = 1; man = 2); age (65–84 years = 1; 30–44 years and 44–64 years = 2); education (low = 1; middle and high = 2); annual income for period 1 and 2 (0–84000 SEK= 1; > 85000 SEK= 2); annual income for period 3 (0–146000 = 1; > 147000 SEK= 2); and migration status (born in Sweden = 1, born in another Nordic, European or non-European country=2).

**Selection proportions for the outcome variable**

Out of our two outcome variables, we limited the bias analysis to financial-related unmet oral care needs (FUOCN) as this outcome was likely affected by the reform, as well as by selection bias by sociodemographic indicators.

Data on the participation rates by those individuals with and without FUOCN was not available, and hence we applied the analysis to two scenarios, based on previous research ([Dissing et al. 2017](#_ENREF_1)): 1) the participation rate was the same among individuals with and without FUOCN, indicating no selection bias by the outcome; 2) the participation rate among individuals with FUOCN was ten percentage points (pp) lower compared to those without FUOCN (-5pp for those with FUOCN and +5pp for those without FUOCN), indicating large selection bias by the outcome. These proportions were applied to the observed data under each assumption. Thus, selection-corrected estimates of the relationship between the participation rate by the explanatory variables (social indicators) and by the outcome (FUOCN) were calculated. From these estimates, it was possible to examine the potential impact of the assumed selection bias.

**Results**

Appendix Table 1 display the results of the bias analysis. Under the assumption of no selection bias by the outcome, the bias-adjusted point estimates were identical or very close to the observed estimates. Under the assumption of considerable selection bias by the outcome, the bias-adjusted estimates were consistently larger for all estimates, and for the weak gender estimates changed direction, from men to women being the disadvantaged group. The degree of selection bias was larger for the post-reform periods than in the pre-reform period.

Taken together, these analyses suggest that the observed magnitude of inequities to the disfavor of socially disadvantaged groups may be underestimated as a consequence of selection bias, particularly during the post-reform periods which had lower participation rate. However, the degree of underestimation seems to be contingent on the presence and degree of the unobserved selection bias by the outcome. As a consequence of the period dependent degree of selection bias, the reported increases in inequities from pre- to post-reform periods likely represent underestimations of the true increase in the magnitude of inequities.

**Appendix Table 1.** Observed and selection bias adjusted measures of the relationship between the participation by each of the social indicators and reporting financial-related unmet oral care needs.

| NO BIAS | Period 1 | | | Period 2 | | | Period 3 | | |
| --- | --- | --- | --- | --- | --- | --- | --- | --- | --- |
|  | RR observed | RR missing | RR adjusted | RR observed | RR missing | RR adjusted | RR observed | RR missing | RR adjusted |
| Gender | 0.92  (0.86, 0.98) | 0.91 | 0.92 | 0.92  (0.86, 0.98) | 0.94 | 0.93 | 0.97  (0.91, 1.03) | 0.97 | 0.97 |
| Age | 2.10  (1.86, 2.37) | 2.08 | 2.09 | 2.17  (1.97, 2.39) | 2.17 | 2.17 | 1.88  (1.73, 2.03) | 1.88 | 1.88 |
| Education | 1.21  (1.13, 1.29) | 1.24 | 1.22 | 1.18  (1.11, 1.26) | 1.20 | 1.19 | 1.43  (1.34, 1.52) | 1.43 | 1.43 |
| Income | 2.11  (1.94, 2.29) | 2.08 | 2.10 | 2.27  (2.11, 2.44) | 2.30 | 2.29 | 2.38  (2.22, 2.55) | 2.41 | 2.40 |
| Migration | 1.88  (1.69, 2.09) | 1.83 | 1.86 | 2.24  (2.09, 2.4) | 2.24 | 2.24 | 2.13  (1.98, 2.30) | 2.13 | 2.13 |
|  |  |  |  |  |  |  |  |  |  |
| 10% BIAS |  |  |  |  |  |  |  |  |  |
| Gender | 0.92  (0.86, 0.98) | 1.28 | 1.09 | 0.92  (0.86, 0.98) | 1.36 | 1.16 | 0.97  (0.91, 1.03) | 1.46 | 1.28 |
| Age | 2.10  (1.86, 2.37) | 2.91 | 2.44 | 2.17  (1.97, 2.39) | 3.08 | 2.65 | 1.88  (1.73, 2.03) | 2.80 | 2.46 |
| Education | 1.21  (1.13, 1.29) | 1.72 | 1.42 | 1.18  (1.11, 1.26) | 1.71 | 1.47 | 1.43  (1.34, 1.52) | 2.11 | 1.85 |
| Income | 2.11  (1.94, 2.29) | 2.88 | 2.50 | 2.27  (2.11, 2.44) | 3.37 | 2.97 | 2.38  (2.22, 2.55) | 3.77 | 3.36 |
| Migration | 1.88  (1.69, 2.09) | 2.45 | 2.18 | 2.24  (2.09, 2.4) | 3.14 | 2.79 | 2.13  (1.98, 2.30) | 3.19 | 2.87 |

Numbers are rate ratios (RR) and RR with 95% confidence intervals (CI).

**Appendix Table 2.** **Comparison of total and financial-related unmet oral care needs across study periods and social indicators**

|  | Total unmet oral care needs | | Financial-related unmet oral care needs | |
| --- | --- | --- | --- | --- |
|  | Model 1^a^  PR (95% CI) | Model 2^b^  PR (95% CI) | Model 1^a^  PR (95% CI) | Model 2^b^  PR (95% CI) |
| Period |  |  |  |  |
| Pre–reform | Ref | Ref | Ref | Ref |
| Early post–reform | 0.81 (0.78, 0.83) | 0.86 (0.83, 0.89) | 0.74 (0.71, 0.77) | 0.82 (0.78, 0.85) |
| Late post–reform | 0.73 (0.71, 0.76) | 0.90 (0.87, 0.93) | 0.62 (0.60, 0.65) | 0.83 (0.79, 0.87) |
|  |  |  |  |  |
| Social indicator |  |  |  |  |
| Gender |  |  |  |  |
| man | Ref | Ref | Ref | Ref |
| woman | 0.99 (0.96, 1.02) | 0.86 (0.84, 0.89) | 1.07 (1.03, 1.11) | 0.89 (0.85, 0.92) |
|  |  |  |  |  |
| Age (years) |  |  |  |  |
| 65–84 | Ref | Ref | Ref | Ref |
| 45–64 | 1.53 (1.46, 1.60) | 1.79 (1.71, 1.87) | 1.70 (1.60, 1.80) | 2.09 (1.97, 2.23) |
| 24–44 | 2.20 (2.11, 2.30) | 2.54 (2.42, 2.65) | 2.65 (2.50, 2.80) | 3.19 (3.00, 3.39) |
|  |  |  |  |  |
| Education |  |  |  |  |
| High | Ref | Ref | Ref | Ref |
| Low | 1.19 (1.16, 1.23) | 1.34 (1.30, 1.38) | 1.29 (1.24, 1.34) | 1.46 (1.41, 1.53) |
|  |  |  |  |  |
| Income |  |  |  |  |
| High | Ref | Ref | Ref | Ref |
| Low | 1.76 (1.71, 1.82) | 1.76 (1.70, 1.81) | 2.35 (2.26, 2.46) | 2.29 (2.19, 2.39) |
|  |  |  |  |  |
| Migration |  |  |  |  |
| Native | Ref | Ref | Ref | Ref |
| Immigrant | 1.85 (1.79, 1.92) | 1.75 (1.69, 1.81) | 1.97 (1.88, 2.06) | 1.83 (1.75, 1.91) |

Numbers are prevalence ratios (PR) with 95% confidence intervals (CI).

^a^ Model 1=crude

^b^ Model 2=adjusted for five social indicators.

**Appendix Table 3. Prevalence ratios (PR) with 95% CI of total and financial-related unmet oral care needs and illustration of how inequities change (%) in the three study periods in 48 intersectional strata of 98,177 participants in the Swedish Health on Equal Terms surveys (2004–2018).**

|  | Total unmet oral care needs | | | | | | | Financial-related unmet oral care needs | | | | | | |
| --- | --- | --- | --- | --- | --- | --- | --- | --- | --- | --- | --- | --- | --- | --- |
|  | Pre-reform  PR (95%CI) | Early post-reform  PR (95%CI) | Late post- reform  PR (95%CI) | P2 vs. P1 (%) | P3 vs. P2 (%) | P3 vs. P1 (%) | Overall change | Pre-reform  PR  (95%CI) | Early post-reform  PR  (95%CI) | Late post- reform  PR (95%CI) | P2 vs. P1 (%) | P3 vs. P2 (%) | P3 vs. P1 (%) | Overall change |
| HiInc, HiEd, Swe, 65–84 years, men | Ref | Ref | Ref | 1.0 | 1.0 | 1.0 | No change | Ref | Ref | Ref | 1.0 | 1.0 | 1.0 | No change |
| HiInc, HiEd, Swe, 65–84 years, women | 1.11  (0.5, 2.51) | 1.16 (0.71, 1.89) | 0.89  (0.62, 1.28) | 4.1 | -23.2 | -20.0 | Decrease | 1.67 (0.61, 4.61) | 1.24 (0.59, 2.61) | 1.05 (0.56, 1.96) | -25.8 | -15.3 | -37.1 | Decrease |
| HiInc, HiEd, Swe, 45–64 years, men | 1.72  (1.06, 2.78) | 1.71 (1.23, 2.37) | 1.94  (1.52, 2.48) | -0.8 | 13.5 | 12.6 | Delayed increase | 1.63 (0.81, 3.25) | 1.78 (1.07, 2.96) | 2.62 (1.69, 4.05) | 9.5 | 47.0 | 61.0 | Persistent increase |
| HiInc, HiEd, Swe, 45–64 years, women | 2.32  (1.44, 3.74) | 1.87 (1.35, 2.59) | 1.77  (1.39, 2.26) | -19.5 | -5.4 | -23.9 | Decrease | 2.75 (1.40, 5.42) | 2.17 (1.32, 3.57) | 2.64 (1.72, 4.04) | -21.1 | 21.5 | -4.1 | Decrease |
| HiInc, HiEd, Swe, 24–44 years, men | 3.06  (1.91, 4.90) | 2.43 (1.76, 3.35) | 2.83  (2.23, 3.58) | -20.6 | 16.4 | -7.6 | Decrease | 3.30 (1.68, 6.48) | 2.98 (1.82, 4.88) | 4.29 (2.81, 6.53) | -9.7 | 43.7 | 29.9 | Delayed increase |
| HiInc, HiEd, Swe, 24–44 years, women | 3.43  (2.13, 5.51) | 3.27 (2.38, 4.49) | 2.91  (2.30, 3.69) | -4.6 | -10.8 | -15.0 | Decrease | 4.18 (2.13, 8.20) | 4.00 (2.46, 6.51) | 5.09 (3.36, 7.72) | -4.3 | 27.3 | 21.8 | Delayed increase |
| HiInc, HiEd, Im, 65–84 years, men | 0.91  (0.13, 6.51) | 1.49 (0.68, 3.22) | 2.35  (1.41, 3.92) | 63.6 | 58.4 | 159.0 | Persistentincrease | Not estimated | 1.98 (0.68, 5.74) | 1.43 (0.44, 4.68) | - | -27.9 | - | - |
| HiInc, HiEd, Im, 65–84 years, women | Not estimated | 1.54 (0.57, 4.14) | 1.63  (0.84, 3.16) | - | 5.8 | - | - | Not estimated | 1.79 (0.43, 7.54) | 2.93 (1.14, 7.51) | - | 63.2 | - | - |
| HiInc, HiEd, Im, 45–64 years, men | 5.09  (2.82, 9.19) | 3.52 (2.39, 5.19) | 3.88  (2.86, 5.26) | -30.9 | 10.2 | -23.8 | Decrease | 5.09 (2.14, 12.14) | 4.90 (2.78, 8.64) | 6.57 (3.98, 10.85) | -3.8 | 34.1 | 28.9 | Delayed increase |
| HiInc, HiEd, Im, 45–64 years, women | 4.64  (2.43, 8.85) | 3.26 (2.21, 4.80) | 3.79  (2.84, 5.07) | -29.9 | 16.5 | -18.3 | Decrease | 5.31 (2.13, 13.24) | 5.26 (3.03, 9.14) | 7.19 (4.48, 11.54) | -0.9 | 36.8 | 35.6 | Delayed increase |
| HiInc, HiEd, Im, 24–44 years, men | 3.98  (1.84, 8.59) | 4.65 (3.16, 6.84) | 5.30  (3.94, 7.12) | 16.8 | 14.0 | 33.2 | Persistentincrease | 3.98 (1.28, 12.36) | 5.09 (2.79, 9.27) | 9.81 (6.04, 15.93) | 27.9 | 92.8 | 146.6 | Persistent increase |
| HiInc, HiEd, Im, 24–44 years, women | 4.26  (2.09, 8.70) | 4.53 (3.07, 6.70) | 4.69  (3.48, 6.33) | 6.4 | 3.5 | 10.1 | Persistentincrease | 6.82 (2.76, 16.85) | 6.08 (3.41, 10.84) | 9.34 (5.79, 15.09) | -10.9 | 53.7 | 37.0 | Delayed increase |
| HiInc, LoEd, Swe, 65–84 years, men | 1.39  (0.70, 2.73) | 1.15  (0.73, 1.80) | 1.82  (1.35, 2.44) | -17.3 | 58.6 | 31.1 | Delayed increase | 1.39 (0.52, 3.67) | 0.95 (0.45, 1.99) | 2.34 (1.40, 3.91) | -31.6 | 146.7 | 68.8 | Delayed increase |
| HiInc, LoEd, Swe, 65–84 years, women | 1.34  (0.58, 3.13) | 1.21  (0.7, 2.09) | 1.38  (0.95, 2.01) | -10.1 | 14.4 | 2.9 | Delayed increase | 1.53 (0.48, 4.88) | 1.16 (0.49, 2.75) | 1.81 (0.96, 3.42) | -24.4 | 56.4 | 18.2 | Delayed increase |
| HiInc, LoEd, Swe, 45–64 years, men | 2.28  (1.41, 3.70) | 2.53  (1.83, 3.49) | 2.63  (2.06, 3.34) | 10.8 | 3.8 | 15.0 | Persistentincrease | 2.71 (1.37, 5.37) | 2.98 (1.82, 4.89) | 4.48 (2.94, 6.82) | 10.0 | 50.2 | 65.2 | Persistent increase |
| HiInc, LoEd, Swe, 45–64 years, women | 2.41  (1.44, 4.04) | 2.11  (1.49, 3.00) | 2.44  (1.88, 3.16) | -12.5 | 15.4 | 1.0 | Delayed increase | 3.60 (1.77, 7.31) | 3.13 (1.87, 5.26) | 4.40 (2.84, 6.84) | -13.0 | 40.7 | 22.4 | Delayed increase |
| HiInc, LoEd, Swe, 24–44 years, men | 4.15(2.57, 6.70) | 4.07  (2.91, 5.69) | 3.46  (2.53, 4.74) | -2.0 | -14.8 | -16.5 | Decrease | 5.53 (2.81, 10.89) | 5.63 (3.39, 9.34) | 8.28 (5.14, 13.34) | 1.8 | 47.0 | 49.6 | Persistent increase |
| HiInc, LoEd, Swe, 24–44 years, women | 6.99 (4.31, 11.33) | 3.66  (2.46, 5.44) | 4.99 (3.52, 7.06) | -47.6* | 36.2 | -28.7 | Decrease | 12.34 (6.29, 24.18) | 6.31 (3.62, 11.00) | 10.91 (6.47, 18.39) | -48.8 | 72.7 | -11.6 | Decrease |
| HiInc, LoEd, Im, 65–84 years, men | Not estimated | 4.20  (2.24, 7.91) | 2.59 (1.25, 5.37) | - | -38.4 | - | – | Not estimated | 6.87 (3.00, 15.72) | 2.40 (0.58, 9.90) | - | -65.1 | - | - |
| HiInc, LoEd, Im, 65–84 years, women | Not estimated | 2.09 (0.68, 6.38) | 4.25 (2.33, 7.73) | - | 103.3 | - | – | Not estimated | 1.63 (0.22, 11.8) | 6.88 (2.74, 17.29) | - | 323.5 | - | - |
| HiInc, LoEd, Im, 45–64 years, men | 5.50 (2.74, 11.04) | 5.63 (3.84, 8.26) | 5.93 (4.27, 8.23) | 2.4 | 5.3 | 7.8 | Persistentincrease | 7.70 (3.04, 19.50) | 9.03 (5.20, 15.70) | 10.44 (6.13, 17.78) | 17.4 | 15.6 | 35.6 | Persistent increase |
| HiInc, LoEd, Im, 45–64 years, women | 5.01  (2.18, 11.5) | 4.89 (3.13, 7.64) | 3.48 (2.24, 5.40) | -2.4 | -28.9 | -30.6 | Decrease | 5.01 (1.45, 17.36) | 7.61 (4.07, 14.23) | 5.13 (2.51, 10.46) | 51.8 | -32.6 | 2.2 | Reboun-ding |
| HiInc, LoEd, Im, 24–44 years, men | 5.22 (2.16, 12.63) | 5.57 (3.42, 9.08) | 6.34 (3.81, 10.56) | 6.7 | 13.9 | 21.5 | Persistentincrease | 8.36 (2.81, 24.82) | 9.58 (4.96, 18.5) | 14.23 (7.03, 28.8) | 14.6 | 48.6 | 70.3 | Persistent increase |
| HiInc, LoEd, Im, 24–44 years, women | 2.61 (0.40, 17.25) | 10.08 (6.46, 15.73) | 9.73 (6.20, 15.28) | 286.1 | -3.5 | 272.7 | Reboun–ding | 5.22 (0.75, 36.47) | 18.57 (10.08, 34.23) | 24.77 (13.33, 46.05) | 255.6 | 33.4 | 374.4 | Persistent increase |
| LoInc, HiEd, Swe, 65–84 years, men | 2.43 (1.41, 4.18) | 2.41 (1.66, 3.50) | 2.45  (1.86, 3.23) | -0.8 | 1.7 | 0.8 | Little change | 3.81 (1.83, 7.93) | 3.20 (1.84, 5.57) | 4.70 (2.98, 7.42) | -16.0 | 47.0 | 23.5 | Delayed increase |
| LoInc, HiEd, Swe, 65–84 years, women | 1.28 (0.69, 2.36) | 1.14 (0.74, 1.74) | 1.44 (1.07, 1.93) | -11.2 | 26.9 | 12.7 | Delayed increase | 1.46 (0.62, 3.43) | 1.74 (0.96, 3.17) | 2.47 (1.53, 3.98) | 19.2 | 41.5 | 68.7 | Persistent increase |
| LoInc, HiEd, Swe, 45–64 years, men | 4.31 (2.67, 6.96) | 3.94 (2.77, 5.61) | 4.13 (3.07, 5.54) | -8.5 | 4.6 | -4.2 | Decrease | 5.69 (2.89, 11.23) | 6.31 (3.77, 10.58) | 8.73 (5.46, 13.97) | 10.9 | 38.3 | 53.4 | Persistent increase |
| LoInc, HiEd, Swe, 45–64 years, women | 3.33 (2.09, 5.33) | 2.69 (1.94, 3.75) | 3.21 (2.47, 4.17) | -19.2 | 19.1 | -3.8 | Decrease | 5.12 (2.64, 9.93) | 4.46 (2.73, 7.28) | 8.13 (5.30, 12.46) | -12.9 | 82.4 | 58.9 | Delayed increase |
| LoInc, HiEd, Swe, 24–44 years, men | 5.74 (3.63, 9.08) | 4.99 (3.64, 6.84) | 5.04 (3.96, 6.41) | -13.1 | 1.0 | -12.2 | Decrease | 8.63 (4.48, 16.59) | 8.35 (5.19, 13.46) | 10.77 (7.11, 16.31) | -3.1 | 28.9 | 24.9 | Delayed increase |
| LoInc, HiEd, Swe, 24–44 years, women | 4.82 (3.06, 7.60) | 4.64 (3.43, 6.30) | 4.34 (3.45, 5.45) | -3.7 | -6.6 | -10.0 | Decrease | 7.50 (3.92, 14.38) | 8.21 (5.16, 13.08) | 9.45 (6.32, 14.13) | 9.4 | 15.1 | 25.9 | Persistent increase |
| LoInc, HiEd, Im, 65–84 years, men | 6.09 (2.82, 13.15) | 4.07 (2.50, 6.62) | 4.73 (3.27, 6.83) | -33.2 | 16.1 | -22.4 | Decrease | 6.96 (2.31, 20.98) | 8.14 (4.36, 15.19) | 11.26 (6.62, 19.15) | 16.9 | 38.4 | 61.7 | Persistent increase |
| LoInc, HiEd, Im, 65–84 years, women | 1.99 (0.49, 8.01) | 2.94 (1.76, 4.91) | 2.87 (1.85, 4.44) | 47.8 | -2.5 | 44.1 | Reboun–ding | 3.98 (0.92, 17.27) | 5.06 (2.57, 9.94) | 5.65 (2.99, 10.69) | 27.1 | 11.9 | 42.1 | Persistent increase |
| LoInc, HiEd, Im, 45–64 years, men | 9.18 (5.42, 15.54) | 8.38 (5.96, 11.79) | 8.17 (6.14, 10.89) | -8.7 | -2.5 | -10.9 | Decrease | 8.23 (3.67, 18.47) | 15.74 (9.55, 25.95) | 17.79 (11.17, 28.33) | 91.3 | 13.0 | 116.2 | Persistent increase |
| LoInc, HiEd, Im, 45–64 years, women | 7.54 (4.47, 12.71) | 6.43 (4.58, 9.04) | 6.47 (4.88, 8.59) | -14.6 | 0.6 | -14.1 | Decrease | 12.49 (6.12, 25.5) | 10.06 (6.06, 16.70) | 14.6 (9.26, 23.03) | -19.5 | 45.1 | 16.9 | Delayed increase |
| LoInc, HiEd, Im, 24–44 years, men | 11.21 (6.84, 18.36) | 7.56 (5.40, 10.58) | 6.23 (4.65, 8.35) | -32.6 | -17.5 | -44.4  * | Decrease | 18.34 (9.20, 36.58) | 13.13 (7.98, 21.63) | 13.75 (8.62, 21.94) | -28.4 | 4.7 | -25.0 | Decrease |
| LoInc, HiEd, Im, 24–44 years, women | 6.96 (4.24, 11.44) | 6.55 (4.75, 9.02) | 6.58 (5.10, 8.49) | -6.0 | 0.5 | -5.5 | Decrease | 10.86 (5.44, 21.68) | 11.24 (6.94, 18.2) | 14.77 (9.63, 22.67) | 3.5 | 31.4 | 36.1 | Persistent increase |
| LoInc, LoEd, Swe, 65–84 years, men | 2.99 (1.86, 4.82) | 2.64 (1.90, 3.66) | 3.20 (2.52, 4.06) | -11.7 | 21.2 | 7.0 | Delayed increase | 4.09 (2.09, 8.03) | 3.71 (2.27, 6.08) | 6.59  (4.37, 9.96) | -9.3 | 77.7 | 61.1 | Delayed increase |
| LoInc, LoEd, Swe, 65–84 years, women | 2.43 (1.51, 3.91) | 2.04  (1.48, 2.81) | 2.36  (1.87, 2.99) | -16.1 | 15.9 | -2.8 | Decrease | 2.89 (1.47, 5.69) | 2.44 (1.49, 3.98) | 4.58  (3.03, 6.91) | -15.8 | 87.9 | 58.2 | Delayed increase |
| LoInc, LoEd, Swe, 45–64 years, men | 4.93 (3.11, 7.82) | 5.10 (3.71, 7.00) | 7.01 (5.51, 8.91) | 3.4 | 37.4 | 42.1 | Persistentincrease | 7.31 (3.79, 14.09) | 8.47 (5.25, 13.66) | 17.21  (11.4, 25.97) | 15.9 | 103.2  * | 135.5 | Persistent increase |
| LoInc, LoEd, Swe, 45–64 years, women | 3.56 (2.25, 5.64) | 3.45 (2.53, 4.72) | 5.02  (3.97, 6.35) | -3.1 | 45.3 | 40.8 | Delayed increase | 5.39 (2.80, 10.36) | 5.85 (3.64, 9.39) | 11.65  (7.75, 17.53) | 8.6 | 99.3  * | 116.4 | Persistent increase |
| LoInc, LoEd, Swe, 24–44 years, men | 7.11 (4.48, 11.28) | 6.40 (4.58, 8.94) | 7.79 (5.87, 10.34) | -10.0 | 21.7 | 9.5 | Delayed increase | 11.28 (5.86, 21.73) | 11.60 (7.08, 19.00) | 20.39 (13.06, 31.85) | 2.8 | 75.8 | 80.7 | Persistent increase |
| LoInc, LoEd, Swe, 24–44 years, women | 6.53 (4.12, 10.34) | 6.45 (4.68, 8.87) | 7.65 (5.89, 9.93) | -1.3 | 18.6 | 17.1 | Delayed increase | 10.49 (5.46, 20.18) | 11.84 (7.33, 19.11) | 21.46 (14.04, 32.78) | 12.8 | 81.2 | 104.5 | Persistent increase |
| LoInc, LoEd, Im, 65–84 years, men | 6.71 (3.33, 13.55) | 5.77 (3.91, 8.52) | 5.80 (4.18, 8.05) | -14.1 | 0.5 | -13.7 | Decrease | 10.44 (4.20, 25.95) | 9.48 (5.43, 16.56) | 13.48 (8.20, 22.15) | -9.2 | 42.1 | 29.0 | Delayed increase |
| LoInc, LoEd, Im, 65–84 years, women | 4.76 (2.47, 9.19) | 4.49 (3.08, 6.54) | 4.65 (3.42, 6.31) | -5.8 | 3.5 | -2.5 | Decrease | 7.33 (3.11, 17.26) | 7.53 (4.40, 12.90) | 10.04 (6.21, 16.23) | 2.8 | 33.2 | 36.9 | Persistent increase |
| LoInc, LoEd, Im, 45–64 years, men | 11.94 (7.31, 19.49) | 9.52 (6.82, 13.30) | 9.97 (7.57, 13.15) | -20.2 | 4.7 | -16.4 | Decrease | 17.90 (8.94, 35.88) | 18.31 (11.19, 29.96) | 21.23 (13.41, 33.62) | 2.3 | 16.0 | 18.6 | Persistent increase |
| LoInc, LoEd, Im, 45–64 years, women | 7.39 (4.45, 12.27) | 7.26 (5.20, 10.12) | 8.06  (6.13, 10.6) | -1.8 | 11.1 | 9.1 | Delayed increase | 9.96 (4.87, 20.36) | 12.24 (7.46, 20.10) | 20.52 (13.22, 31.87) | 22.9 | 67.6 | 106.0 | Persistent increase |
| LoInc, LoEd, Im, 24–44 years, men | 8.55 (4.81, 15.17) | 10.31 (7.25, 14.67) | 12.09 (9.07, 16.11) | 20.7 | 17.2 | 41.4 | Persistentincrease | 16.14 (7.66, 34.01) | 18.05 (10.75, 30.32) | 31.97 (20.30, 50.34) | 11.8 | 77.1 | 98.0 | Persistent increase |
| LoInc, LoEd, Im, 24–44 years, women | 9.79 (5.82, 16.48) | 9.19 (6.56, 12.87) | 7.90  (5.70, 10.96) | -6.2 | -14.0 | -19.3 | Decrease | 15.01 (7.28, 30.96) | 16.02 (9.71, 26.42) | 18.74 (11.39, 30.81) | 6.7 | 17.0 | 24.8 | Persistent increase |

P1 = pre-reform period, P2 = early post-reform period, P3 = late post-reform period

Ref = reference group, HiInc = high income, HiEd = high education, LoInc = low income, LoEd = low education, Swe = Sweden–born, Im = immigrant.

*Significant comparison tested by interaction effects (p<0.05)

**Apendix Table 4. Level of missing information for total and financial-related unmet oral care needs (UOCN) across social indicators in the three study periods.**

|  | Pre-reform | | | | Early post-reform | | | | Late post- reform | | | |
| --- | --- | --- | --- | --- | --- | --- | --- | --- | --- | --- | --- | --- |
|  | Total UOCN | | Financial-related  UOCN | | Total  UOCN | | Financial-related  UOCN | | Total UOCN | | Financial-related  UOCN | |
|  | Not missing  N(%) | Missing  N(%) | Not missing  N(%) | Missing  N(%) | Not missing  N(%) | Missing  N(%) | Not missing  N(%) | Missing  N(%) | Not missing  N(%) | Missing  N(%) | Not missing  N(%) | Missing  N(%) |
| Total sample |  |  |  |  |  |  |  |  |  |  |  |  |
| Gender |  |  |  |  |  |  |  |  |  |  |  |  |
| Man | 10723 (99.1) | 101 (0.9) | 10723 (99.1) | 101 (0.9) | 15253 (98.8) | 177 (1.2) | 15253 (98.9) | 177 (1.1) | 18681 (98.8) | 232 (1.2) | 18681 (98.8) | 232 (1.2) |
| Woman | 12903 (98.9) | 144 (1.1) | 12903 (98.9) | 144 (1.1) | 18725 (98.4) | 305 (1.6) | 18725 (98.4) | 305 (1.6) | 21892 (98.6) | 327  (1.4) | 21892 (98.5) | 327 (1.5) |
| Education |  |  |  |  |  |  |  |  |  |  |  |  |
| High | 12705 (99.4) | 80 (0.6) | 12705 (99.4) | 80 (0.6) | 19508 (99.2) | 162 (0.8) | 19508 (99.2) | 162 (0.8) | 24855 (99.3) | 177 (0.7) | 24855 (99.3) | 177 (0.7) |
| Low | 10921 | 165 (1.5) | 10921 (98.5) | 165 (1.5) | 14470 (97.8) | 320 (2.2) | 14470 (97.8) | 320 (2.2) | 15718 (97.6) | 382  (2.4) | 15718 (97.6) | 382 (2.4) |
| Age (years) |  |  |  |  |  |  |  |  |  |  |  |  |
| 65–84 | 3882 (97.4) | 103 (2.6) | 3882 (97.4) | 103 (2.6) | 8413 (97.0) | 260 (3.0) | 8413 (97.0) | 260 (3.0) | 13718 (97.4) | 370 (2.6) | 13718 (97.4) | 370 (2.7) |
| 45–64 | 10309 (99.1) | 88 (0.9) | 10309 (99.2) | 88 (0.8) | 14257 (99.0) | 149 (1.0) | 14257 (99.0) | 149 (1.0) | 15601 (99.1) | 144 (0.9) | 15601 (99.1) | 144 (0.9) |
| 24–44 | 9435 (99.4) | 54 (0.6) | 9435 (99.4) | 54 (0.6) | 11308 (99.4) | 73 (0.6) | 11308 (99.4) | 73 (0.6) | 11254 (99.6) | 45 (0.4) | 11254 (99.6) | 45 (0.4) |
| Income |  |  |  |  |  |  |  |  |  |  |  |  |
| High | 8540 (99.3) | 64 (0.7) | 15900 (99.2) | 128 (0.8) | 15900 (99.2) | 128 (0.8) | 15900 (99.2) | 128 (0.8) | 22431 (99.2) | 179 (0.8) | 22431 (99.2) | 179 (0.8) |
| Low | 15086 (98.8) | 181 (1.2) | 18078 (98.1) | 354 (1.9) | 18078 (98.1) | 354 (1.9) | 18078 (98.1) | 354 (1.9) | 18142 (98.0) | 380 (2.0) | 18142 (97.9) | 380 (2.1) |
| Immigrant |  |  |  |  |  |  |  |  |  |  |  |  |
| No | 22387 (99.0) | 228 (1.0) | 22387 (99.0) | 228 (1.0) | 29098 (98.7) | 385 (1.3) | 29098 (98.7 ) | 385 (1.3) | 35570 (98.6) | 497 (1.4) | 35570 (98.6) | 497 (1.4) |
| Yes | 1239 (98.6) | 17 (1.4) | 1239 (98.7) | 17 (1.3) | 4880 (98.1) | 97 (1.9) | 4880 (98.1) | 97 (1.9) | 5003 (98.8) | 62 (1.2) | 5003 (98.8) | 62 (1.2) |

**Appendix Figure 1. Change in relative inequities of unmet oral care needs for 48 intersectional strata in three study periods.**


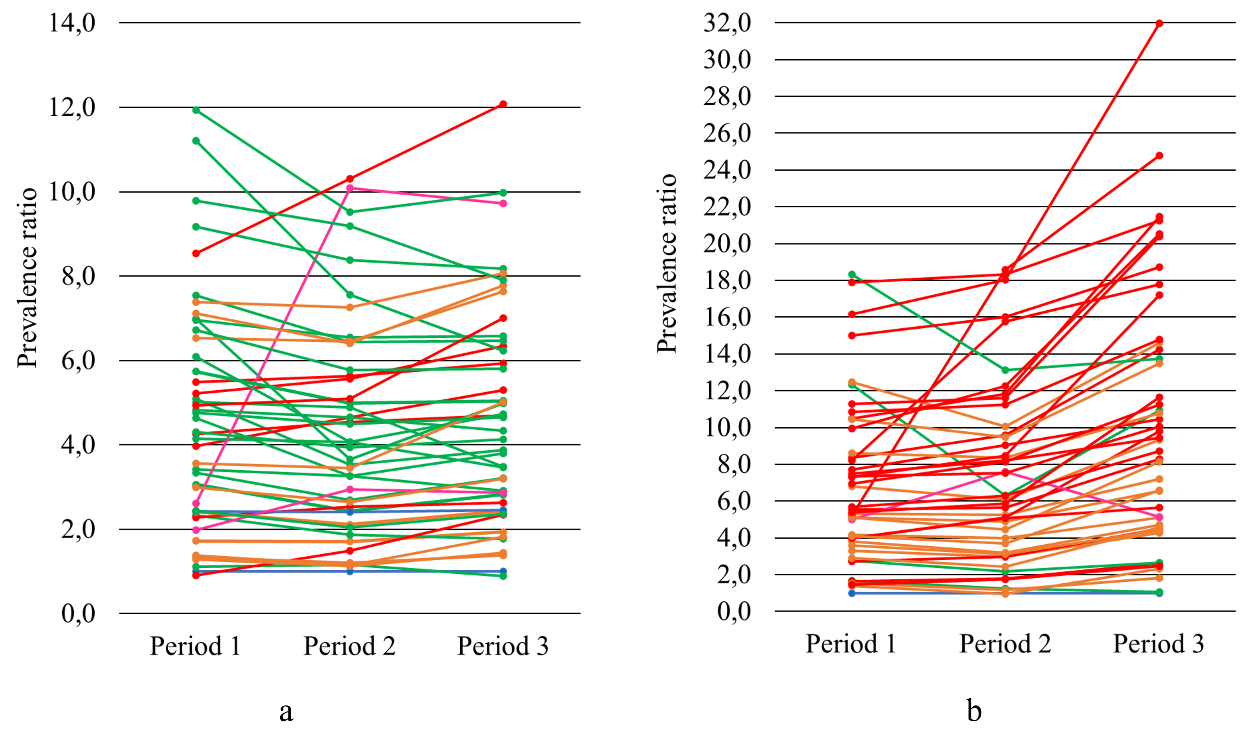


a: Total unmet oral care needs. b: Financial–related unmet oral care needs.

Points represent prevalence ratios of total and financial-related unmet oral care needs, with native men aged >65 years with high education and high income as the reference stratum.

**Appendix Figure 2. Area under the curve (AUC) for total and financial-related unmet oral care needs by year and three study periods.**

**
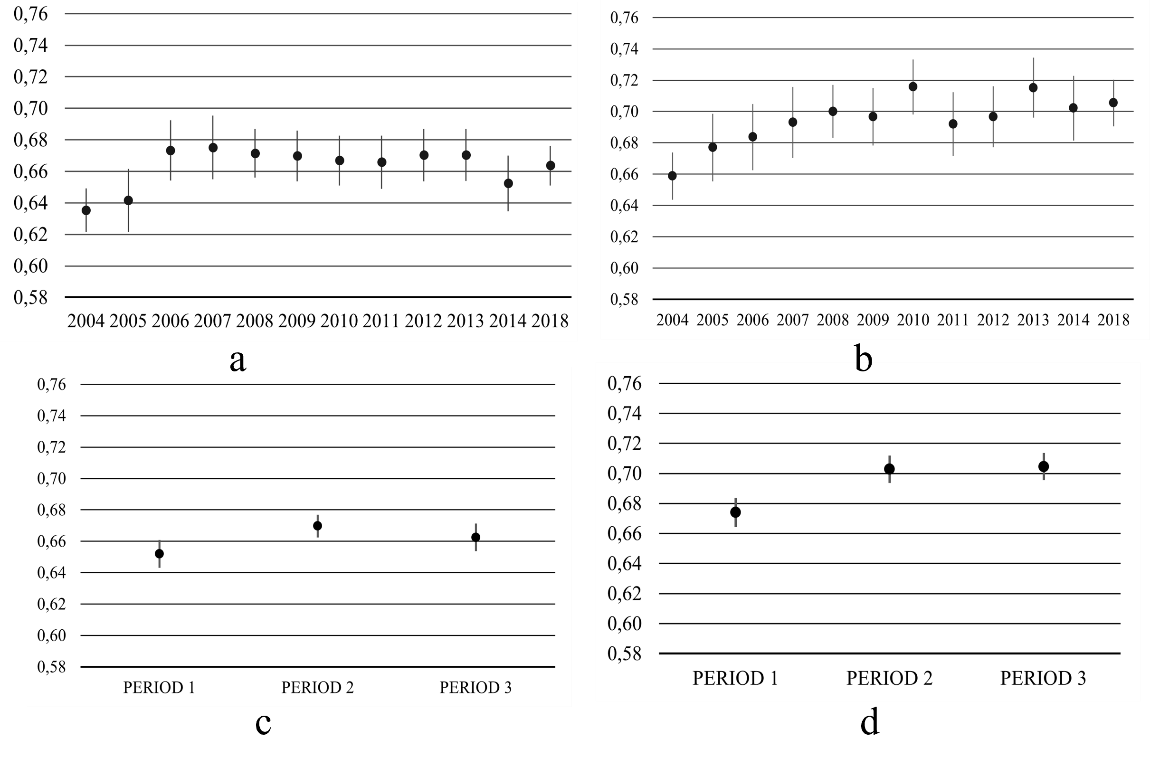
**

a:Total unmet oral care needs by year. b: Financial-related unmet oral care needs by year.

c: Total unmet oral care needs by period. d: Financial-related unmet oral care needs by period.

Estimates represent AUC values and 95% confidence intervals

**References**

Dissing AS, Dich N, Andersen AN, Lund R, Rod NH. 2017. Parental break-ups and stress: Roles of age & family structure in 44 509 pre-adolescent children. Eur J Public Health. 27(5):829-834.

Fox MP, MacLehose RF, Lash TL. 2021. Applying quantitative bias analysis to epidemiologic data. Springer.

Riksrevisionen. 2012. Tandvårdsreformen 2008 – når den alla? Stockholm.

Statens Offentliga Utredningar. 2021. När behovet får styra – ett tandvårdssystem för en mer jämlik tandhälsa.

Statistiska Centralbyrån. 2004 Hälsa på lika vilkor?Enkätundersökning 2004, det nationella urvalet teknisk rapport <https://www.folkhalsomyndigheten.se/contentassets/9b1b216c596a487ca6c6aa6dc413efb4/teknisk-rapport-med-bilagor-hlv-2004.pdf>.

Statistiska Centralbyrån. 2010. Hälsa på lika vilkor? Enkätundersökning 2010, nationellt urval tekninsk rapport. <https://www.folkhalsomyndigheten.se/contentassets/9b1b216c596a487ca6c6aa6dc413efb4/teknisk-rapport-med-bilagor-hlv-2010.pdf>.

Statistiska Centralbyrån. 2018. Teknisk rapport en beskrivning av genomförande och metoder "hälsa på lika vilkor" nationellt urval 2028-08-31. <https://www.folkhalsomyndigheten.se/contentassets/9b1b216c596a487ca6c6aa6dc413efb4/teknisk-rapport-med-bilagor-hlv-2018.pdf>

**STROBE Statement—Checklist of items that should be included in reports of *cross-sectional studies***

|  | Item No | Recommendation | Page(s); line(s) |
| --- | --- | --- | --- |
| **Title and abstract** | 1 | (*a*) Indicate the study’s design with a commonly used term in the title or the abstract | 1 |
|  |  | (*b*) Provide in the abstract an informative and balanced summary of what was done and what was found | 2–3 |
| Introduction | | |  |
| Background/rationale | 2 | Explain the scientific background and rationale for the investigation being reported | 4–5; 76–130 |
| Objectives | 3 | State specific objectives, including any prespecified hypotheses | 6, 128–130 |
| Methods | | |  |
| Study design | 4 | Present key elements of study design early in the paper | 6; 133–142 |
| Setting | 5 | Describe the setting, locations, and relevant dates, including periods of recruitment, exposure, follow-up, and data collection | 6; 133–142 |
| Participants | 6 | (*a*) Give the eligibility criteria, and the sources and methods of selection of participants | 6–7, 146–152 |
| Variables | 7 | Clearly define all outcomes, exposures, predictors, potential confounders, and effect modifiers. Give diagnostic criteria, if applicable | 7–8; 154–177 |
| Data sources/ measurement | 8* | For each variable of interest, give sources of data and details of methods of assessment (measurement). Describe comparability of assessment methods if there is more than one group | 7–8; 154–177 |
| Bias | 9 | Describe any efforts to address potential sources of bias | Appendix File 5 |
| Study size | 10 | Explain how the study size was arrived at | 6–7; 146–152 |
| Quantitative variables | 11 | Explain how quantitative variables were handled in the analyses. If applicable, describe which groupings were chosen and why | 7; 168–173 |
| Statistical methods | 12 | (*a*) Describe all statistical methods, including those used to control for confounding | 8–9; 179–213 |
|  |  | (*b*) Describe any methods used to examine subgroups and interactions | 8–9; 188–200 |
|  |  | (*c*) Explain how missing data were addressed | 6–7; 148–150 |
|  |  | (*d*) If applicable, describe analytical methods taking account of sampling strategy | Not applicable |
|  |  | (*e*) Describe any sensitivity analyses | Appendix File 4 and Appendix Figure 2 |
| Results | | |  |
| Participants | 13* | (a) Report numbers of individuals at each stage of study—eg numbers potentially eligible, examined for eligibility, confirmed eligible, included in the study, completing follow–up, and analysed | 6–7; 146–152 |
|  |  | (b) Give reasons for non-participation at each stage | Not applicable |
|  |  | (c) Consider use of a flow diagram | Not applicable |
| Descriptive data | 14* | (a) Give characteristics of study participants (eg demographic, clinical, social) and information on exposures and potential confounders | Table 1 |
|  |  | (b) Indicate number of participants with missing data for each variable of interest | Appendix Table 4 |
| Outcome data | 15* | Report numbers of outcome events or summary measures | Table 1 |
| Main results | 16 | (*a*) Give unadjusted estimates and, if applicable, confounder-adjusted estimates and their precision (eg, 95% confidence interval). Make clear which confounders were adjusted for and why they were included | Appendix Table 1, Table 2 |
|  |  | (*b*) Report category boundaries when continuous variables were categorized | Not applicable |
|  |  | (*c*) If relevant, consider translating estimates of relative risk into absolute risk for a meaningful time period | Not applicable |
| Other analyses | 17 | Report other analyses done—eg analyses of subgroups and interactions, and sensitivity analyses | 11–12; 263–286  Table 3.  Appendix File 4 and Appendix Figure 2 |
| Discussion | | |  |
| Key results | 18 | Summarise key results with reference to study objectives | 12; 294–300 |
| Limitations | 19 | Discuss limitations of the study, taking into account sources of potential bias or imprecision. Discuss both direction and magnitude of any potential bias | 16; 378–387.  Appendix File 5. |
| Interpretation | 20 | Give a cautious overall interpretation of results considering objectives, limitations, multiplicity of analyses, results from similar studies, and other relevant evidence | 12–15; 301–349 |
| Generalisability | 21 | Discuss the generalisability (external validity) of the study results | 15, 374–376 |
| Other information | | |  |
| Funding | 22 | Give the source of funding and the role of the funders for the present study and, if applicable, for the original study on which the present article is based | 17; 397–398 |

*Give information separately for exposed and unexposed groups.

**Note:** An Explanation and Elaboration article discusses each checklist item and gives methodological background and published examples of transparent reporting. The STROBE checklist is best used in conjunction with this article (freely available on the Web sites of PLoS Medicine at http://www.plosmedicine.org/, Annals of Internal Medicine at http://www.annals.org/, and Epidemiology at http://www.epidem.com/). Information on the -STROBE Initiative is available at www.strobe-statement.org.
