# Supplementary material for: Inequities in Unmet Oral Care Needs after a Swedish Subsidization Reform: An Intersectional Analysis
Source: JDR Clin Trans Res. 2024 Dec 19;10(4):416–26. doi: 10.1177/23800844241305109 (PMC12402522; doi:10.1177/23800844241305109)
Supplement: sj-pptx-1-jct-10.1177_23800844241305109 – Supplemental material for Inequities in Unmet Oral Care Needs after a Swedish Subsidization Reform: An Intersectional Analysis [file sj-pptx-1-jct-10.1177_23800844241305109.pptx]

## Slide 1
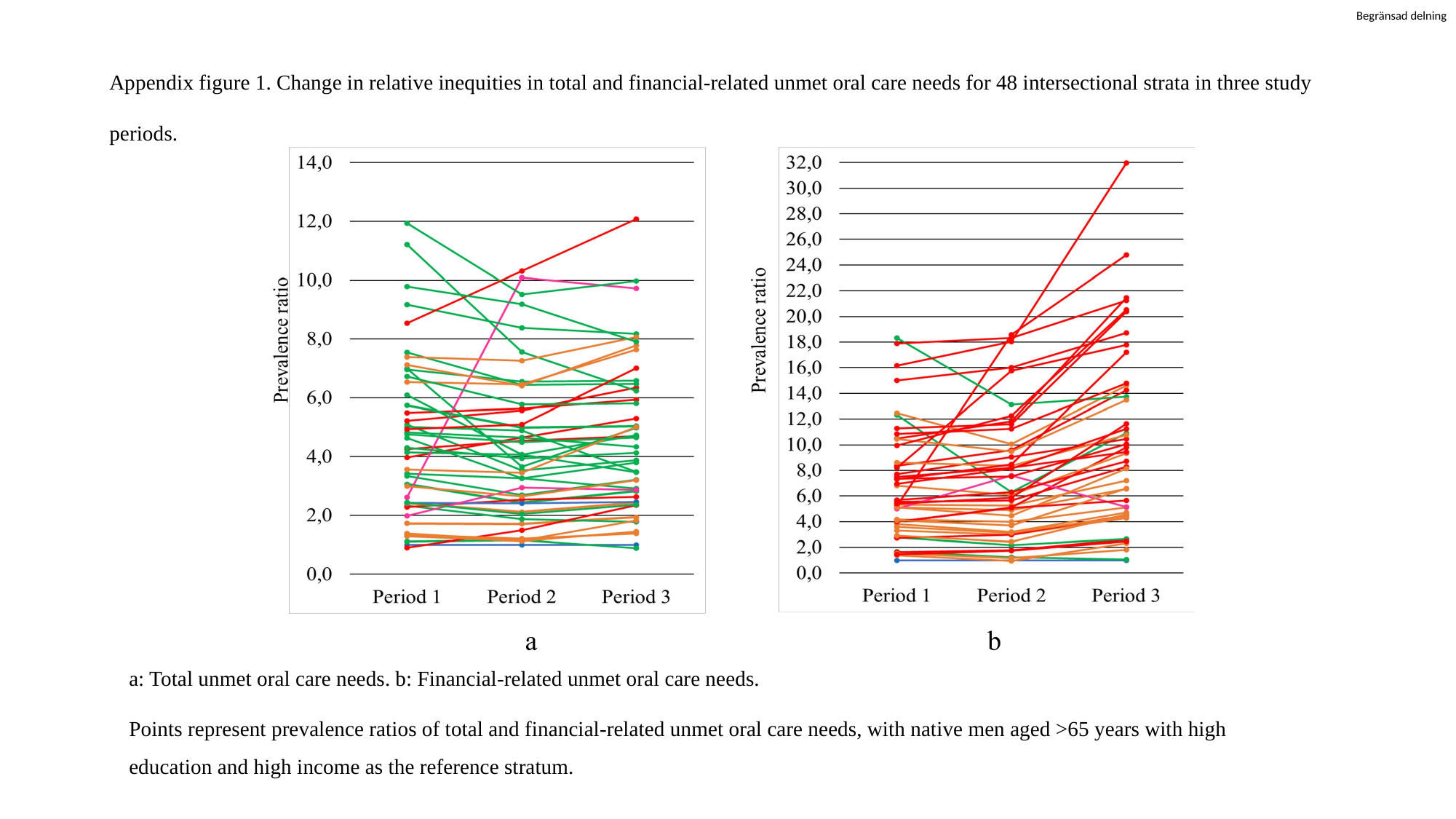

Appendix figure 1. Change in relative inequities in total and financial-related unmet oral care needs for 48 intersectional strata in three study periods.
a: Total unmet oral care needs. b: Financial-related unmet oral care needs.
Points represent prevalence ratios of total and financial-related unmet oral care needs, with native men aged >65 years with high education and high income as the reference stratum.
