# Supplementary material for: Inequities in Unmet Oral Care Needs after a Swedish Subsidization Reform: An Intersectional Analysis
Source: JDR Clin Trans Res. 2024 Dec 19;10(4):416–26. doi: 10.1177/23800844241305109 (PMC12402522; doi:10.1177/23800844241305109)
Supplement: sj-pptx-2-jct-10.1177_23800844241305109 – Supplemental material for Inequities in Unmet Oral Care Needs after a Swedish Subsidization Reform: An Intersectional Analysis [file sj-pptx-2-jct-10.1177_23800844241305109.pptx]

## Slide 1
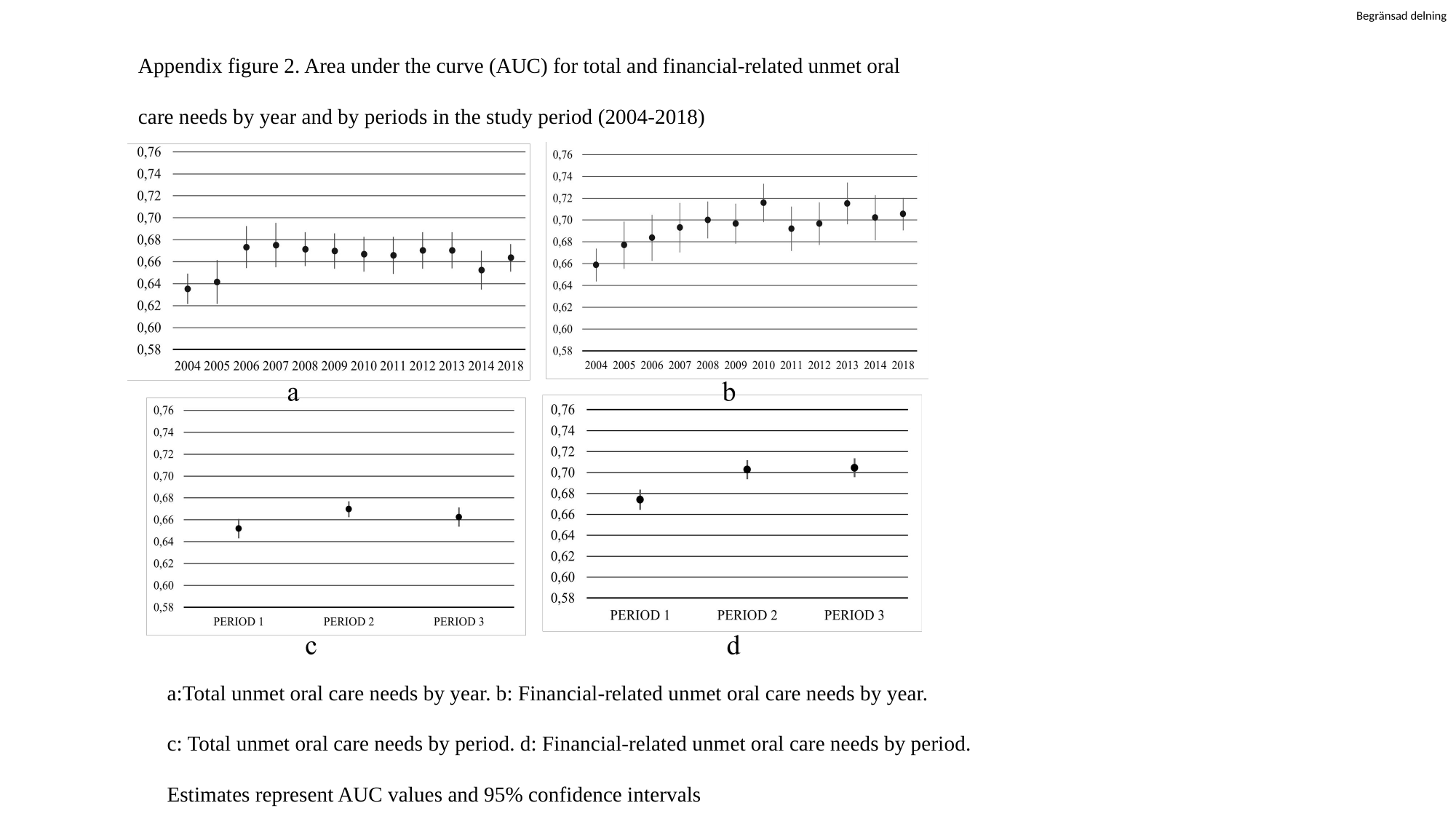

Appendix figure 2. Area under the curve (AUC) for total and financial-related unmet oral care needs by year and by periods in the study period (2004-2018)
a:Total unmet oral care needs by year. b: Financial-related unmet oral care needs by year.
c: Total unmet oral care needs by period. d: Financial-related unmet oral care needs by period.
Estimates represent AUC values and 95% confidence intervals
